# Reproductive Decision‐Making Among BRCA1/2 Pathogenic Variants Carriers and Physicians: Attitudes Toward Preimplantation and Prenatal Genetic Testing

**DOI:** 10.1002/pd.70111

**Published:** 2026-03-02

**Authors:** Marta D'Alonzo, Silvia Actis, Giulia Lavalle, Simona Suraci, Luca Pace, Valentina Elisabetta Bounous, Annamaria Ferrero

**Affiliations:** ^1^ Department of Surgical Sciences Gynaecology and Obstetrics Unit Mauriziano Umberto I Hospital University of Turin Turin Italy

## Abstract

**Objective:**

The objective of this paper is to investigate the knowledge and attitudes of BRCA1/2 pathogenic variant carriers and gynecologists regarding preimplantation genetic tests for monogenic diseases (PGT‐M) and prenatal diagnosis (PND).

**Methods:**

A survey using structured questionnaires was completed by 89 BRCA1/2 carriers and 37 gynecologists, assessing awareness, attitudes, and ethical concerns.

**Results:**

Before receiving information, only 28% of carriers were aware of PGT‐Mand PND. Once informed, the majority believed that these options should be discussed with all BRCA mutation carriers as part of standard reproductive counseling (87.6% for PGT‐M and 85.7% for PND). Many indicated a willingness to consider their use. Nonetheless, concerns remained, particularly regarding hormonal stimulation, pregnancy termination, and ethical implications. Among gynecologists, awareness of PGT‐M and PND as options for BRCA carriers was inconsistent (45.9% and 51.4%, respectively), and few routinely discussed these options with patients. Ethical concerns were common, and 86.5% of clinicians expressed the need for clearer guidelines and multidisciplinary collaboration.

**Conclusion:**

The findings reveal a discrepancy between reproductive preferences of BRCA mutation carriers and current practices and knowledge among healthcare professionals. Bridging this gap will require educational efforts, development of guidelines, and a multidisciplinary approach to reproductive counseling in the context of hereditary cancer risk.

## Highlights

- What is already known about this topic?
  ◦ BRCA1/2 mutations confer high cancer risk and a 50% chance of transmission to offspring.
  ◦ Preimplantation genetic tests for monogenic diseases (PGT‐M) and prenatal diagnosis (PND) are available but remain ethically debated.
  ◦ Previous studies show limited awareness among carriers and inconsistent counseling by physicians.
  ◦ The uptake of PGT‐M/PND among BRCA carriers has historically been low, despite conceptual support.
- What does this study add?
  ◦ Demonstrates a strong interest in PGT‐M/PND among Italian BRCA carriers once informed.
  ◦ Reveals major concerns about hormonal stimulation and pregnancy termination.
  ◦ Highlights physicians' limited awareness, Ethical reservations, and low rates of counseling.
  ◦ The dual‐perspective design highlights a striking discrepancy between patient expectations and physician awareness and counseling practices, stressing the need for guidelines and multidisciplinary counseling.

## 1 Introduction

Hereditary Breast and Ovarian Cancer (HBOC) syndrome is an inherited condition characterized by an increased risk of breast and ovarian cancer. Most HBOCs are caused by mutations in the BRCA1 or the BRCA2 gene (BRCA1/2m) [1, 2].

Patients with these mutations face a significantly higher lifetime risk of developing breast cancer, with estimates ranging from 60% to 72% for BRCA1 and 55%–69% for BRCA2, compared to 12.3% in the general population. Additionally, BRCA1/2 mutations increase the risk of ovarian cancer (39%–58% for BRCA1 and 13%–29% for BRCA2), pancreatic cancer (up to 5% for BRCA1 and 5%–10% for BRCA2), and prostate cancer (7%–26% for BRCA1 and 19%–61% for BRCA2) [3, 4, 5, 6, 7, 8, 9].

Advances in genetic testing have resulted in more people discovering their BRCA1/2 carrier status before or during their reproductive years. As awareness increases, so does the need for comprehensive reproductive counseling that considers medical, ethical, and psychosocial factors.

HBOC has incomplete penetrance, meaning that not all individuals who inherit a pathogenic variant will develop the associated cancers. Offspring of mutation carriers have a 50% chance of inheriting the mutation, and the clinical presentation of the syndrome can vary among affected family members. For individuals concerned about transmitting the mutation to their children, reproductive counseling is recommended. This counseling offers a comprehensive evaluation of the risks, benefits, and limitations of available reproductive options, including prenatal genetic diagnosis (PND) and preimplantation genetic test for monogenic diseases (PGT‐M), the latter requiring in vitro fertilization, whereas PND can be performed in any ongoing pregnancy, including spontaneous conceptions [10].

PND involves the genetic analysis of chorionic villi (typically performed between 10 and 13 weeks of gestation) or amniotic fluid (usually from 15 weeks onward), allowing parents to make informed reproductive decisions, including the possibility of pregnancy termination [11] PGT‐M is performed on embryos created through in vitro fertilization (IVF). This process tests a few cells at an early developmental stage [11, 12]. This approach enables the selection of embryos that do not carry the mutation for implantation, which may reduce but does not eliminate the likelihood of considering pregnancy termination, as confirmatory prenatal testing is often recommended. However, IVF may not always result in a successful pregnancy.

Ethical guidelines from organizations such as the European Society of Human Reproduction and Embryology (ESHRE) and the American Society for Reproductive Medicine (ASRM) consider PGT‐M ethically acceptable for adult‐onset conditions, including hereditary breast and ovarian cancer syndrome (HBOC syndrome), while emphasizing case‐by‐case evaluation [13, 14].

However, the application of these reproductive technologies continues to be a subject of debate within both scientific and ethical circles, raising concerns among affected individuals and healthcare professionals alike [14]. Dominant autosomal inheritance, incomplete penetrance, and late onset of HBOC further complicate these discussions [15].

This study aims to evaluate the knowledge and attitudes of BRCA1/2 mutation carriers, as well as the clinicians involved in their counseling and clinical management.

## 2 Materials and Methods

This study was conducted on adult patients with BRCA1 and BRCA2 gene mutations who attended the BRCA Outpatient Clinic at the A.O. Ordine Mauriziano Umberto I Breast Unit in Turin, alongside physicians involved in their clinical management.

The study protocol was approved by the Institutional Ethics Committee of Mauriziano Umberto I Hospital (Protocol No. 52044, 9 May 2022; No. 231/2022), and all participants provided informed consent before taking part in the study.

Eligible mutation carriers included both women of reproductive age and post‐reproductive carriers.

Medical Genetics, Oncology, and Gynecology/Obstetrics clinicians from Turin and other hospitals within the Piemonte healthcare network were invited to participate in the study.

Specially developed questionnaires were given to female BRCA mutation carriers and doctors.

For patients, data collection took place during outpatient visits or, for those without scheduled appointments, through telephone or email communication. Physician questionnaires were sent exclusively by email, with a link provided for online completion.

The patient questionnaire consisted of four sections:

1. Medical history: age, mutation type (BRCA1/2), age at carrier diagnosis, personal and family oncological history, preventive surgical procedures, family and marital status, education, and occupation.
2. Awareness and knowledge of BRCA mutation: evaluated patients' understanding of their mutation status, associated risks, and preventive measures through multiple‐choice questions.
3. Attitudes toward PGT‐M and PND: explored participants' perspectives on reproductive choices, including the acceptability of abortion based on fetal sex. A brief informative section on PGT‐M and PND was included. While necessary to ensure informed responses, this may have influenced participants' stated willingness to consider these technologies. Questions regarding prenatal diagnosis focused specifically on chorionic villus sampling (CVS) in order to standardize responses across participants. Data on amniocentesis were not separately collected.
4. Impact on reproductive choices: assessed how carrier status affects reproductive decisions using a scale from 1 (“not at all”) to 4 (“totally”).

The physician questionnaire was structured into three sections:

1. Demographic and professional information: speciality, age, and experience in specialized centers for PGT‐M and PND.
2. Knowledge and clinical practice: evaluated familiarity with PGT‐M and PND, and confidence in recommending these options to BRCA mutation carriers.
3. Attitudes and ethical perspectives: examined clinical perspectives and ethical considerations concerning these reproductive techniques.

### 2.1 Statistical Analysis

Data analysis was performed using IBM SPSS (version 1.0.0.1213). The description of variables was as follows: qualitative variables were expressed as absolute values and percentages, whereas quantitative variables were presented as medians and interquartile ranges.

## 3 Results

### 3.1 BRCA Mutation Carriers' Awareness and Attitudes Toward Reproductive Options

A total of 118 women with a BRCA mutation were invited to participate in the study, of whom 89 provided consent and completed the questionnaire. At the time of data collection, participants ranged in age from 21 to 74 years, with a mean age of 49 years (SD 13). Of these, 52 (58%) carried a pathogenic BRCA1 mutation (BRCA1m), while the remaining 37 (42%) carried a pathogenic BRCA2 mutation (BRCA2m). Overall, 57 women (64%) had at least one child. Forty‐two (47%) participants had a personal history of cancer, including 24 with breast cancer, 15 with ovarian cancer, 2 with both breast and ovarian cancer, and 1 with endometrial cancer. Additionally, 75 participants (84%) reported a family history of cancer.

In terms of risk‐reducing surgery, 53 women had undergone at least one prophylactic procedure. Twelve had a prophylactic mastectomy only, 18 had a prophylactic salpingo‐oophorectomy only, one had a hysterectomy with salpingo‐oophorectomy, and 22 had both.

Regarding marital status, 17 women were single, 60 were married or cohabiting, and 12 were divorced.

Characteristics of the study population are summarized in Table 1.

**TABLE 1:** Characteristics of the population of women.

Characteristic	Value
Total participants	89
Mean age (SD)	49 (SD 13)
BRCA mutation	
BRCA1 carriers	52 (58%)
BRCA2 carriers	37 (42%)
Children	
Participants with children	57 (64%)
Participants without children	32 (36%)
Personal history of cancer	
Yes	42 (47%)
No	47 (53%)
Type of cancer	
Breast cancer	24 (27%)
Ovarian cancer	15 (17%)
Both breast and ovarian cancer	2 (2%)
Endometrial cancer	1 (1%)
No previous personal history of cancer	47 (53%)
Family history of cancer	
Yes	75 (84%)
No	14 (16%)
Risk reducing surgery	
Both mastectomy and salpingo‐oophorectomy	22 (25%)
Prophylactic salpingo‐oophorectomy only	18 (20%)
Prophylactic mastectomy only	12 (14%)
Hysterectomy with salpingo‐oophorectomy	1 (1%)
No prophylactic surgery	36 (40%)

Awareness of the risks associated with the BRCA mutation was investigated. Only one participant (1.1%) reported having little awareness of the risks associated with the BRCA mutation, while the vast majority (98.9%) considered themselves well or moderately informed.

The question about perceived awareness of the risks associated with the BRCA mutation was followed by five additional items assessing participants' knowledge of key aspects of being a BRCA mutation carrier. These included the estimated risk of developing cancer, with 85.4% answering correctly, BRCA‐related malignancies, correctly identified by 82.0%, the incidence of breast cancer in men with a BRCA mutation, with 70.8% responding correctly, and the probability of passing the mutation to offspring, accurately answered by 86.5% of participants. Overall, participants demonstrated a high level of awareness, with knowledge gaps primarily observed regarding the cancer risk conferred to male carriers.

In terms of awareness of reproductive choices, only 25 women (28%) were aware of the possibilities regarding PGT‐M or prenatal diagnosis (PND), while the remaining 64 (72%) were not. The mean age did not differ between informed and uninformed patients (48.9 and 49.4 years).

Participants were asked to answer a series of questions after reading a short paragraph about PGT‐M and PND.

#### 3.1.1 Preimplantation Genetic Testing for Monogenic Disorders (PGT‐M)

In the overall population, 87.6% (78 out of 89) of participants believed that the possibility of undergoing PGT‐M should be offered to all BRCA mutation carriers. Additionally, 73.0% (65 out of 89) stated they would have used or would consider using PGT‐M if it had been available.

Among the 56 participants who had children, 39 (69.6%) said they would have used PGT‐M if it had been available. However, 26 of them (66.7%) expressed concerns about the hormonal stimulation required for the procedure. Conversely, among the 17 women who would not have chosen PGT‐M, 10 (58.8%) cited hormone stimulation as their primary concern.

Among the 33 participants without children, 26 (78.8%) stated they would consider using PGT‐M before pregnancy if it were available. Of these, 20 (76.9%) were concerned about the hormonal stimulation involved. All participants who would not opt for PGT‐M cited concerns about hormonal stimulation.

Overall, 70.8% of all participants (63 out of 89) expressed concerns about hormonal stimulation associated with PGT‐M, regardless of whether they would have chosen to undergo the procedure or not.

#### 3.1.2 Prenatal Diagnosis (PND)

Regarding prenatal diagnosis (PND) through chorionic villus sampling, 85.4% (77 out of 89) of respondents believed that the option of undergoing PND should be made available to all BRCA mutation carriers.

Among the 56 participants with children, 48 (85.7%) supported offering PND. Among these, 38 (67.9%) said they would or would have considered undergoing PND. However, if the fetus tested positive for a BRCA mutation, only 14 (36.8%) would have opted for pregnancy termination.

Among the 33 participants without children, 29 (87.9%) believed that PND should be available to BRCA carriers. Of these, 25 (86.2%) would consider using the test. However, only 5 (20%) would choose to terminate the pregnancy if the fetus tested positive for a BRCA mutation.

Looking at the entire group, 19 participants had either previously undergone or would consider pregnancy termination if the fetus carried the BRCA mutation. Among them, 16 (84.2%) said they would make this decision regardless of the fetus's sex, whereas 3 (15.8%) would opt for selective termination only if the fetus were female.

Finally, two participants reported having chosen not to have children due to their BRCA mutation. Both stated that they would have reconsidered their decision if PGT‐M and PND had been available to them.

Participants were also asked: “*Based on your experience, do you believe that the quality of life of a BRCA mutation carrier justifies selection through the previously mentioned methods?*” A total of 45 (50.6%) participants responded affirmatively.

Regarding decision‐making, 64 (71.9%) women stated that they would discuss this choice with their partner, while the remaining participants considered it a personal decision.

Additionally, several questions were asked to assess the impact of BRCA mutation awareness on reproductive choices. Participants rated their responses on a scale from 1 to 4 (1 = *not at all*, 4 = *completely*):

- Conflicts with partner: 61 *not at all*, 5 *slightly*, 3 *moderately*, 1 *very much.*
- Fear of passing the mutation to children: 27 *not at all*, 8 *slightly*, 16 *moderately*, 19 *very much.*
- Concern about pregnancy‐induced cancer onset: 29 *not at all*, 11 *slightly*, 14 *moderately*, 16 *very much.*
- Fear of inadequate cancer surveillance during pregnancy: 39 *not at all*, 10 *slightly*, 13 *moderately*, 9 *very much.*
- Concern about developing cancer at a young age and being unable to care for children as desired: 27 *not at all*, 11 *slightly*, 8 *moderately*, 23 *very much.*

### 3.2 Healthcare Professionals' Perspectives on Reproductive Options for BRCA Mutation Carriers

This section presents an in‐depth analysis of the responses from 37 healthcare professionals, all of whom are gynecologists, regarding their awareness, attitudes, and ethical considerations related to reproductive options for BRCA mutation carriers.

The questionnaire was distributed to gynecologists and other specialists involved in family planning services and multidisciplinary care teams (GICs) across the Turin, Piedmont area in Italy. A total of approximately 157 professionals were invited, resulting in a response rate of 23.6% (37/157).

All respondents were gynecologists, with a significant majority (29 out of 37, 78.4%) reporting that they directly manage BRCA mutation carriers in their clinical practice. In terms of workplace settings, 26 participants (70.3%) stated they work in prenatal diagnosis centers, while 8 (21.6%) were involved in assisted reproduction centers.

Knowledge about the reproductive options available to BRCA mutation carriers was heterogeneous. Less than half (17 respondents, 45.9%) were aware that PGT‐M could be an option for these patients. In comparison, a slightly higher percentage (19 respondents, 51.4%) were familiar with the possibility of offering prenatal diagnosis (PND). The discussion on pregnancy termination in the case of a BRCA‐positive fetus was even less well‐known, with only 14 professionals (37.8%) stating that they were aware of the ongoing debate.

The potential impact of BRCA mutations on fertility remains a controversial topic among clinicians. When asked whether BRCA mutations could be associated with a reduced ovarian reserve, only 8 participants (21.6%) believed this to be the case, whereas the majority (29, 78.4%) disagreed. Similarly, only 10 respondents (27%) considered that BRCA mutations might affect ovarian response to hormonal stimulation, whereas the remaining 27 (73%) did not perceive an influence. These findings suggest a lack of consensus on the reproductive implications of BRCA mutations, which may impact how these issues are discussed with patients.

**FIGURE 1:**
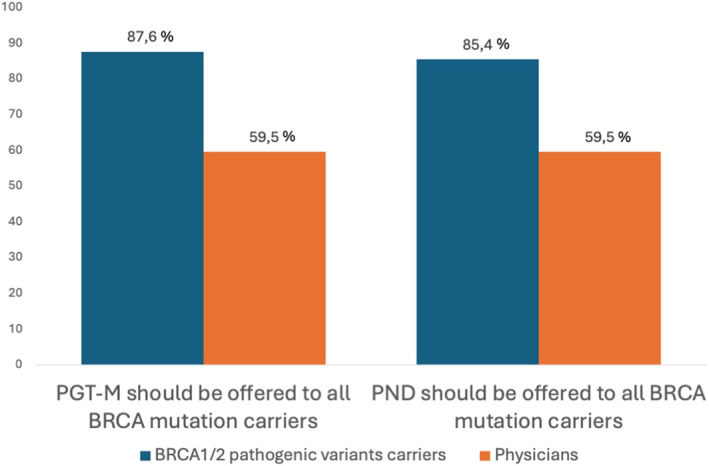
Discrepancy between BRCA carriers' and clinicians' perspectives on reproductive counseling, preimplantation genetic test for monogenic diseases (PGT‐M) and prenatal diagnosis (PND).

When asked about preconception counseling, 59.5% of professionals (22 out of 37) supported the idea that all BRCA carriers should be informed about available reproductive options, including PGT‐M and PND. However, 9 clinicians (24.3%) stated that they would provide this information only upon specific patient request. Only 6 clinicians reported having already discussed PGT‐M and PND with BRCA mutation carriers, despite 29 out of 37 stating that they manage patients with a BRCA mutation. This discrepancy is visually illustrated in Figure 1, which compares the proportion of healthcare professionals and BRCA carriers who believe that reproductive options should be discussed with all mutation carriers. The contrast highlights a significant gap between patient expectations and clinical practice.

The ethical dimension of reproductive selection was also explored. A large proportion of respondents expressed concerns about potential genetic selection and the ethical implications of applying PGT‐M and PND to BRCA carriers. Specifically:

- 18.9% of professionals had low confidence in the idea that scientific progress and improved surveillance make these procedures unnecessary, while 37.8% completely agreed with this statement.
- 43.2% feared that offering these methods could lead to a slippery slope toward 'designer babies'.
- 37.8% were concerned that allowing BRCA‐related selection could spark controversy over the use of genetic testing for well‐established conditions (e.g., cystic fibrosis, SMA).
- 32.4% expressed complete ethical opposition to the use of PGT‐M and PND for BRCA mutations.

These findings reflect diverging ethical stances, with some clinicians seeing PGT‐M/PND as a legitimate reproductive option, whereas others remain skeptical or opposed due to broader ethical and social implications.

#### 3.2.1 The Need for a Multidisciplinary Discussion

Given the ethical complexity and the lack of clear national guidelines, 32 respondents (86.5%) agreed on the need for a multidisciplinary meeting to establish national or regional directives on reproductive options for BRCA mutation carriers. This widespread support for a structured discussion highlights the necessity of developing standardized counseling protocols to guide both clinicians and patients in making informed decisions.

## 4 Discussion

The findings from our study align with previous research on the acceptance of PGT‐M, demonstrating that a significant proportion of BRCA mutation carriers support the availability of reproductive genetic testing. In our cohort, 87.6% believed that PGT‐M should be offered to all BRCA mutation carriers, closely aligning with the 86.2% of women in Dervin et al.'s study who considered that PGT‐M should be proposed to all BRCA mutation carriers, regardless of the severity of their family history [16].

In our study, 85.4% of participants supported offering PND through chorionic villus sampling to all BRCA mutation carriers, a slightly higher rate than previous findings, which reported that 66.7% of BRCA carriers considered PND acceptable [16].

While previous studies [16] reported that only 47.1% of participants considered or would consider PGT‐M for themselves, our study found a substantially higher proportion (73.0%). In addition, in a survey of adequate adherence to the PGT‐M, Mor et al. (2018) found that only 25.7% of BRCA‐positive women who were offered PGT‐M at no cost chose to use it [17]. This finding is comparable to other studies, which report uptake rates ranging from 10% to 26% [18]. Gietel‐Habets et al. highlighted that while 80% of BRCA carriers found PGT‐M acceptable, only 39% would consider using it for themselves [19]. This suggests that while conceptual support for PGT‐M is increasing, significant barriers to utilization remain, including ethical concerns, financial costs, and emotional burdens.

Similarly, our findings on PND uptake demonstrate higher levels of acceptance than prior research. Among participants with children, 67.9% stated that they would or had considered undergoing the procedure. Among those without children, 86.2% would consider using the test. These rates exceed previous reports, such as the 29.9% acceptance of PND found in other studies [16]. However, despite this broad acceptance, the willingness to terminate a pregnancy based on a BRCA mutation diagnosis remains low. In our study, only 36.8% of participants with children and 20% of those without children would consider termination if the fetus tested positive, consistent with prior research where only 20% of carriers supported termination [16]. This reluctance is consistent with previous literature, underscoring the ethical and emotional dilemmas surrounding reproductive choices for BRCA mutation carriers.

Overall, our findings suggest a growing openness toward reproductive genetic testing among BRCA carriers, yet a persistent gap between theoretical support and actual utilization. This discrepancy underscores the need for further investigation into the factors that influence decision‐making, including ethical considerations, access to care, and the psychosocial implications of genetic risk.

Notably, at the time of data collection, none of the BRCA mutation carriers followed at our outpatient clinic had actually undergone PGT‐M or PND, which further emphasises the gap between theoretical acceptance and clinical adoption of these techniques.

One of the interesting predictors for PGT‐M uptake is a history of infertility. Mor et al. reported that BRCA carriers who experienced infertility were nearly 20 times more likely to pursue PGT‐M [17]. Similarly, Zuckerman et al. noted that couples at risk of transmitting genetic conditions frequently opted for PGT‐M only when IVF was already indicated [20]. This suggests that concerns about reproductive difficulties often outweigh genetic risk considerations in the decision‐making process.

The psychological burden associated with PGT‐M also plays a crucial role. Pastore et al. emphasized the significant distress and decisional uncertainty experienced by patients considering PGT‐M [21]. The complexity of the procedure, the need for ovarian stimulation, and the emotional toll of embryo selection can be deterrents. Our findings corroborate these concerns, as 70.8% of our participants expressed apprehension about hormonal stimulation.

Additionally, reproductive decisions among BRCA mutation carriers are influenced by concerns about passing on the mutation and their health risks. Chan et al. found that 41% of BRCA carriers reported that their mutation status affected their decision to have children [22]. This aligns with our findings, where women cited fears about passing on the mutation, pregnancy‐induced cancer risks, and concerns about their ability to care for children given their heightened cancer risk.

One of the most pressing issues identified in this study is the lack of comprehensive reproductive counseling. Despite the availability of PGT‐M and PND, awareness remains suboptimal. Our analysis revealed that only 45.9% of the surveyed gynecologists were aware that PGT‐M was an option for BRCA carriers, and 51.4% were aware of the possibility of offering PND. This limited knowledge is concerning, given that patients rely on their healthcare providers for information and guidance in making reproductive decisions. Improving education and access to genetic counseling could help BRCA carriers make informed reproductive decisions that align with their values and circumstances. The attitudes of physicians toward PGT‐M and PND for BRCA1/2 mutation carriers reflect a combination of ethical considerations, scientific knowledge, clinical experience, and systemic barriers. A critical finding from our study is the heterogeneity in physician knowledge and perspectives.

One of the most critical aspects that emerged was physicians' level of knowledge about PGT‐M. Previous studies have shown that while many specialists are aware of the availability of these techniques, their in‐depth understanding of their applicability to BRCA1/2 carriers is often limited [23]. According to existing literature, only a portion (24%) of specialists actively discuss PGT‐M with their patients, and even fewer provide active referrals to specialized centers [24].

Medical knowledge appears to be directly correlated with clinical practice: professionals who are more informed about PND are more likely to discuss these options with patients and refer them to appropriate centers [24]. Sixty‐three per cent of physicians consider their lack of knowledge about PGT‐M as one of the main barriers to its application [24].

Ethical opinions on PGT‐M and PND for BRCA1/2 mutations are complex. While most professionals believe that PGT‐M is an acceptable option to prevent mutation transmission [24], some raise concerns about the ethical justification for using this technology for a condition with incomplete penetrance and late‐onset [23]. Some physicians hesitate to recommend PGT‐M, believing that the decision should be left entirely to the patients. In contrast, others actively support it as a means of reducing the incidence of hereditary breast and ovarian cancer (HBOC) in future generations [23].

Regarding PND, physicians are divided on the possibility of offering pregnancy termination if the fetus tests positive for a BRCA1/2 mutation. While some consider this option ethically questionable due to the incomplete penetrance of the gene [25], other physicians see it as a reproductive right that should be openly discussed with patients [25].

Despite recognizing the importance of PGT‐M, the number of physicians actively referring patients for this procedure is significantly lower than the number who consider it acceptable [23]. In one study, only 24% of physicians had discussed PGT‐M with their patients, and only 23% had ever referred a patient for the test [24]. Among the main obstacles identified, in addition to limited knowledge, are high costs, lack of clear guidelines, and disparities in access between different healthcare facilities [26].

Moreover, referral disparities vary based on the physician's speciality: obstetrician‐gynecologists appear more likely to discuss and refer patients for PGT‐M compared to internists or pediatricians [24]. This suggests the need for more uniform education across different medical disciplines involved in genetic counseling for BRCA1/2 carriers.

Given these knowledge gaps, ethical concerns, and practical barriers, there is a strong need for more straightforward guidelines and structured discussions on reproductive options for BRCA carriers. The majority of gynecologists in our study (86.5%) supported the establishment of a multidisciplinary meeting to develop national or regional directives on this topic. This widespread agreement underscores the need to develop standardized counseling protocols that integrate genetic, ethical, and reproductive considerations to guide both clinicians and patients in making informed decisions. Addressing these gaps through targeted education, improved access to genetic counseling, and the development of consensus guidelines could help bridge the divide between theoretical support for reproductive genetic testing and its actual clinical application.

While previous studies have explored attitudes toward PGT‐M and PND among BRCA carriers, the novelty of our study lies in the dual‐perspective design within an Italian healthcare context, highlighting a striking discrepancy between patient expectations and physician awareness and counseling practices. Moreover, the Italian legal and healthcare framework, including abortion legislation and public reimbursement policies, adds distinct ethical and practical dimensions not fully explored in prior work.

In Italy, where this study was conducted, access to medically assisted reproduction and PGT‐M within the National Health System varies by region and clinical indication. While some regions offer partial or full reimbursement for assisted reproductive technology (ART) and genetic testing in specific cases, access for healthy BRCA carriers without infertility remains inconsistent. This variability may represent a substantial barrier to uptake, even among motivated carriers, and should be considered when interpreting the gap between conceptual support and actual utilization. Moreover, under Italian law (Law 194/1978), termination of pregnancy is permitted within the first 90 days for social, economic, health, or familial reasons and beyond this timeframe only in cases of serious risk to the woman's life or fetal anomalies. The classification of a BRCA pathogenic variant as a sufficient medical indication for termination is not explicitly defined, potentially contributing to both patient uncertainty and clinician hesitancy. This legal ambiguity may partially explain the low willingness to terminate pregnancies based on BRCA status observed in our study.

This study has several limitations that should be taken into account when interpreting the findings. Firstly, the sample was drawn from a single hospital, which may limit the generalizability of the results to other populations or healthcare settings with different approaches to reproductive counseling for BRCA1/2 mutation carriers. There is also a potential selection bias, as participants were predominantly women with a high level of awareness about their genetic condition, which may not fully represent the broader BRCA1/2 mutation carrier population. Another potential selection bias is represented by the low physician response rate (23.6%), as clinicians with greater interest or stronger opinions on reproductive genetics may have been more likely to participate. Furthermore, although clinicians from medical genetics, oncology, and gynecology/obstetrics were invited, all respondents were gynecologists. The absence of medical geneticists and oncologists may skew the findings, as counseling practices and familiarity with PGT‐M and PND differ substantially by specialty. Moreover, the use of self‐administered questionnaires could have introduced biases due to social desirability or subjective interpretation of the questions, and the short informational section on PGT‐M and PND provided to patients prior to answering several key questions, while necessary for informed participation, might have influenced the high levels of post‐information acceptance.

The patient cohort was older (mean age 49 years), with a high proportion of participants post‐reproductively. This reflects attitudes, values, and perceptions of BRCA1/2 pathogenic variants’ carriers rather than imminent reproductive choices.

Given the limited sample size and the descriptive nature of the study, we did not perform exploratory subgroup analyses, as such analyses would have been potentially misleading. This represents an important direction for future research.

Only female BRCA mutation carriers were included in this study. Male carriers and partners or spouses of carriers represent additional populations whose perspectives may meaningfully influence reproductive decision‐making. Future studies should incorporate these groups.

## 5 Conclusions

This study highlights a significant gap between the reproductive counseling needs of BRCA mutation carriers and the knowledge and preparedness of healthcare providers. While BRCA carriers show strong interest in preimplantation genetic tests for monogenic diseases (PGT‐M) and prenatal genetic diagnosis (PND), their awareness of these options is limited. In contrast, gynecologists demonstrate heterogeneous knowledge and diverging ethical perspectives, with concerns about genetic selection and reproductive decision‐making.

Despite the ethical and emotional complexities, most healthcare professionals recognize the need for standardized guidelines and multidisciplinary discussions to improve counseling practices. Addressing these gaps requires enhanced education for providers, clear national protocols, a patient‐centered approach that considers ethical and psychological concerns, and greater collaboration among specialists. By enhancing reproductive counseling, healthcare professionals can better support BRCA carriers in making informed and personalized reproductive decisions.

## Funding

The authors have nothing to report.

## Ethics Statement

Approval to conduct this research was obtained by the A.O.U. CITTA’ DELLA SALUTE E DELLA SCIENZA DI TORINO – A.O. ORDINE MAURIZIANO DI TORINO – A.S.L. CITTÀ DI TORINO ethics committee, Protocol No. 52044, 9 May 2022; No. 231/2022. All procedures followed were in accordance with the ethical standards of the responsible committee on human experimentation (institutional and national) and with the Helsinki Declaration of 1975, as revised in 2000.

## Consent

Informed consent was obtained from all patients for being included in the study.

## Conflicts of Interest

The authors declare no conflicts of interest.

## Data Availability

The datasets generated and analyzed during the current study are available from the corresponding author on reasonable request.
